# Changes in nonfunctional adrenal incidentaloma after COVID-19 infection and a model for predicting benign and malignant adrenal incidentaloma

**DOI:** 10.3389/fendo.2024.1374282

**Published:** 2024-09-02

**Authors:** Danlei Chen, Sheng Zeng, Qian Liu

**Affiliations:** ^1^ Department of Urology, Tianjin First Central Hospital, Tianjin, China; ^2^ Department of Urology, First People’s Hospital of Yunnan Province, Kunming, China

**Keywords:** adrenal incidentaloma, nonfunctional adrenal incidentaloma, COVID-19, prediction model, malignant potential

## Abstract

**Aims:**

To compare nonfunctional adrenal incidentalomas (NFAI) in individuals with and without a history of COVID-19 infection, while also establishing predictive models for distinguishing between benign and malignant adrenal incidentalomas (AI).

**Methods:**

A retrospective collection of data from patients with AI who underwent surgery and were verified in our hospital between April 2022 and June 2023 was conducted. A total of 121 patients were included in the study. Demographic information, tumor characteristics, functional indicators, and complications were compared among the patients. Statistical analyses utilized the t-test for continuous variables and Pearson chi-square test or Fisher’s exact test for categorical variables.

**Results:**

Patients with COVID-19 exhibited a higher prevalence of obesity (84.2% vs. 63.3%, P=0.048) and elevated direct bilirubin (DBIL) levels (44.1% vs. 19.2%, P=0.043) compared to those without COVID-19. Moreover, patients with Malignant AI, in contrast to Benign AI, showed higher normal total protein (TP) levels (28.8% vs. 57.1%, P=0.016) and larger tumor sizes (20 vs. 32.5mm, P=0.009). Univariate analysis identified low TP (OR=0.303, 95% CI=0.111-0.825, P=0.020) and tumor size (OR=1.045, 95% CI=1.011-1.080, P=0.009) as potential risk factors for multivariate analysis. A predictive model comprising clinical risk factors (tumor size and low TP) demonstrated an AUC of 0.754 (95% CI, 0.603-0.904) with a sensitivity of 0.75 and specificity of 0.775. The calibration curve revealed a bias-corrected AUC of 0.77.

**Conclusion:**

No discernible differences in the clinical manifestations of adrenal incidentalomas were observed between cases with and without a history of COVID-19 infection. However, AI with larger tumor diameters and lower than normal levels of total protein exhibited a more pronounced malignant potential.

## 1 Introduction

Adrenal Incidentalomas (AIs) refer to asymptomatic adrenal masses exceeding 1 cm in diameter, incidentally discovered during imaging studies conducted for reasons unrelated to adrenal pathology (1). The term “adrenal incidentaloma” (AI) was initially proposed by Geelhoed in 1982, who reported 20 related cases (2). The predominant subtype of AI is nonfunctional adrenal incidentaloma (NFAI), constituting 70-85% of cases (3–5). Some researchers contend that small, nonfunctioning adrenal tumors with low CT attenuation may not necessitate intervention or prolonged follow-up (6).

The global spread of Coronavirus Disease 2019 (COVID-19) has manifested with a clinical spectrum ranging from asymptomatic infection to severe disease (7, 8). Notably, the virus exploits the surface-bound peptidase angiotensin-converting enzyme 2 (ACE2) for cellular entry, initiating tissue infection and viral replication (9–12). ACE2, integral to the renin-angiotensin system (RAS), regulates the expression of peptide hormones Ang II and Ang-(1–7), exerting opposing actions through distinct receptors (10, 13–17). Previous studies have associated Ang II with cellular hypertrophy in rat adrenal glomerulosa cells, implicating it in the regulation of long-term steroid metabolism and cell growth/proliferation (18, 19). Additionally, a correlation between AI and insulin resistance has been identified (20).

Emerging evidence suggests that SARS-CoV-2 may target the adrenal glands, raising the prospect of COVID-19 complications being linked to adrenal dysfunction (21). Histopathological examinations indicate structural damage to the adrenal glands, particularly affecting the vascular system, though widespread cellular damage to cortical cells is less likely to lead to an immediate adrenal crisis (21). Persistent cytokine storms, observed in long COVID patients, may be associated with aggregates of SARS-CoV-2 discovered in the adrenal cortex (22).

Our recent findings reveal an increased prevalence of adrenal incidentalomas post the COVID-19 outbreak. While some attribute this rise to the elevated chest CT examination rate, we posit a plausible association with COVID-19. Building on prior research, we propose a conceivable link between COVID-19 infection and AI.

## 2 Method

### 2.1 Patients

**Figure 1:**
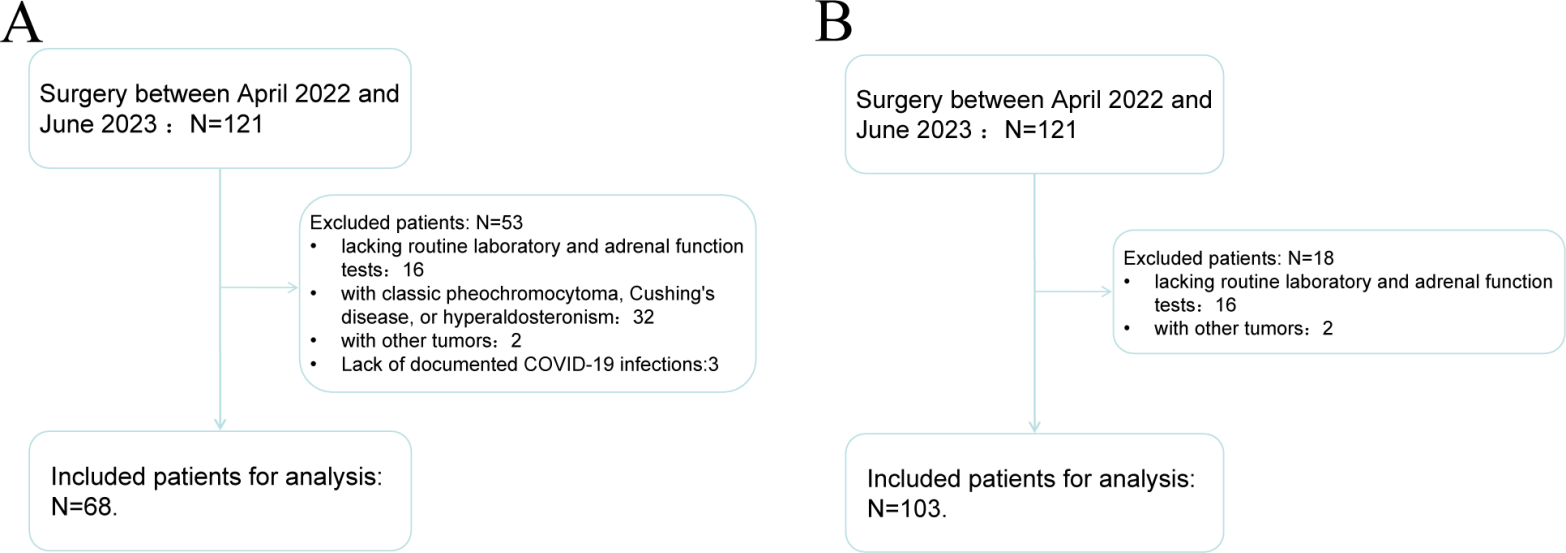
Flow chart of patient screening for COVID-19 & AI study **(A)** and Benign AI & Malignant AI study **(B)**.

The study received approval from the Clinical Research Ethics Committee of Tianjin First Central Hospital, and informed consent was waived due to the retrospective nature of the research. The study adhered to the principles outlined in the Declaration of Helsinki (2013 revision). A comprehensive search of the pathology database identified 121 patients with adrenal incidentalomas who underwent adrenalectomy surgery between April 2022 and June 2023 at our institution. Patients lacking routine laboratory and adrenal function tests (N=16) were excluded, along with 9 patients diagnosed with classic pheochromocytoma, Cushing’s disease, or hyperaldosteronism, and 2 patients with other tumors. Ultimately, 68 patients were included in the study for COVID-19 & AI and 103 patients for Benign AI & Malignant AI (**Figure 1**).

### 2.2 Pathological review

Retrospective review of all pathologic data for the 121 patients was conducted by two experienced pathologists (MF Zhang and XX Guo, with 30 and 28 years of experience in uropathology, respectively). In cases of discordant opinions on pathological slices, both pathologists re-evaluated the slices collaboratively until a consensus was reached. Reviewers were blinded to clinical diagnostic information.

### 2.3 Data collection

Demographic and clinical data were extracted from medical records, encompassing gender, age at onset, preoperative body mass index (BMI), primary tumor location, primary tumor size, and an extensive array of laboratory parameters, including blood cell counts, biochemical markers, and hormonal levels.

### 2.4 Statistical analysis

Statistical analyses were performed using SPSS version 26.0 (IBM SPSS Inc., Chicago, IL, USA) and R software version 4.0.1 (http://www.r-project.org). Continuous variables with normal distribution were presented as mean (standard deviation) and analyzed using Student’s t-test, while non-normally distributed variables were presented as median (interquartile range) and assessed with the Mann-Whitney U test. Categorical variables were compared using the Pearson chi-square test or Fisher’s exact test.

Univariate and multivariate logistic regression analyses were conducted to identify factors associated with COVID-19 in AI and malignant tumors in AI. Variables with P<0.05 in univariate analysis were candidates for multivariate logistic regression analysis, retaining significance in the model. Models were constructed based on identified factors, and their performance was assessed for discrimination and calibration. Discrimination was evaluated by the area under the receiver operating characteristic (ROC) curve (AUC), while calibration was tested by plotting observed and predicted outcome probabilities. A two-sided test with P<0.05 was considered statistically significant.

## 3 Result

### 3.1 COVID-19 & AI

A total of 68 patients were included in the final analysis, categorized as having either COVID-19 (n=38) or non-COVID-19 (n=30). Patients with COVID-19 exhibited a higher prevalence of obesity/abnormal BMI compared to non-COVID-19 patients (84.2% vs. 63.3%, P=0.048) and higher levels of direct bilirubin (DBIL) (44.1% vs. 19.2%, P=0.043). No significant differences were observed in gender, age, blood pressure, tumor size, and other laboratory tests between the two groups. Refer to **Table 1** for a summary of patient characteristics.

**Table 1:** Differences between AI with COVID-19 and AI without COVID-19.

Characteristics	Covid	Non-covid	P value
n	38	30	
Gender, n (%)			0.460
M	16 (42.1%)	10 (33.3%)	
F	22 (57.9%)	20 (66.7%)	
Age, year, mean ± sd	52.184 ± 11.229	54.167 ± 12.793	0.499
Obesity, BMI, n (%)			0.048
Y	32 (84.2%)	19 (63.3%)	
N	6 (15.8%)	11 (36.7%)	
Hypertension, n (%)			0.113
N	13 (34.2%)	16 (53.3%)	
Y	25 (65.8%)	14 (46.7%)	
SIDE, n (%)			0.268
L	23 (60.5%)	22 (73.3%)	
R	15 (39.5%)	8 (26.7%)	
Hemoglobin, n (%)			0.334
Low	7 (20%)	2 (7.4%)	
Normal	26 (74.3%)	24 (88.9%)	
High	2 (5.7%)	1 (3.7%)	
Monocyte, n (%)			0.199
Normal	25 (71.4%)	23 (85.2%)	
High	10 (28.6%)	4 (14.8%)	
Total protein, n (%)			0.820
Low	23 (67.6%)	19 (70.4%)	
Normal	11 (32.4%)	8 (29.6%)	
Albumin, n (%)			0.074
Low	15 (44.1%)	6 (22.2%)	
Normal	19 (55.9%)	21 (77.8%)	
Direct bilirubin, n (%)			0.043
Normal	19 (55.9%)	21 (80.8%)	
High	15 (44.1%)	5 (19.2%)	
Creatinine, n (%)			0.060
Normal	29 (85.3%)	19 (73.1%)	
High	5 (14.7%)	3 (11.5%)	
Low	0 (0%)	4 (15.4%)	
Uric acid, n (%)			0.167
Normal	27 (79.4%)	17 (63%)	
High	7 (20.6%)	8 (29.6%)	
Low	0 (0%)	2 (7.4%)	
K, n (%)			0.244
Low	9 (27.3%)	4 (14.8%)	
Normal	24 (72.7%)	23 (85.2%)	
Prothrombin time, n (%)			0.052
Low	11 (34.4%)	4 (14.8%)	
Normal	21 (65.6%)	20 (74.1%)	
High	0 (0%)	3 (11.1%)	
Thrombin time, n (%)			0.879
Normal	26 (76.5%)	17 (70.8%)	
High	6 (17.6%)	5 (20.8%)	
Low	2 (5.9%)	2 (8.3%)	
ACTH, n (%)			0.524
Normal	24 (82.8%)	18 (78.3%)	
Low	5 (17.2%)	4 (17.4%)	
High	0 (0%)	1 (4.3%)	
Renin (standing), n (%)			0.063
High	8 (25%)	1 (4.2%)	
Low	6 (18.8%)	3 (12.5%)	
Normal	18 (56.2%)	20 (83.3%)	
Renin (decubitus), n (%)			0.884
Normal	23 (69.7%)	19 (73.1%)	
High	2 (6.1%)	2 (7.7%)	
Low	8 (24.2%)	5 (19.2%)	
Tumor Size, median (IQR)	20 (13, 28)	21 (12.75, 30)	0.589

### 3.2 Benign AI & malignant AI

A total of 103 patients were included in the study, categorized as having adrenocortical adenoma (n=79) or pheochromocytoma (PCC) and adrenocortical carcinoma (ACC), the latter exhibiting malignant tendencies. Compared to patients with benign AI, those with malignant AI tended to have higher normal total protein (TP) levels (28.8% vs. 57.1%, P=0.016) and larger tumor sizes (20 vs. 32.5mm, P=0.009) (**Table 2**). No significant differences were observed in gender, age, blood pressure, tumor size, and other laboratory tests between patients with benign AI and malignant AI. Refer to **Table 2** for a summary of patient characteristics.

**Table 2:** Differences between Benign AI and Malignant AI.

Characteristics	Corti-C	Non	P value
n	79	24	
Gender, n (%)			0.257
M	32 (41%)	13 (54.2%)	
F	46 (59%)	11 (45.8%)	
Age, year, median (IQR)	55 (45.5, 61)	58.5 (43.25, 64)	0.559
Obesity, BMI, n (%)			0.513
Y	57 (74%)	17 (81%)	
N	20 (26%)	4 (19%)	
Hypertension, n (%)			0.830
N	31 (40.3%)	9 (42.9%)	
Y	46 (59.7%)	12 (57.1%)	
COVID, n (%)			0.339
1	36 (54.5%)	8 (42.1%)	
0	30 (45.5%)	11 (57.9%)	
SIDE, n (%)			0.318
L	45 (68.2%)	10 (55.6%)	
R	21 (31.8%)	8 (44.4%)	
Monocyte, n (%)			0.774
Normal	60 (81.1%)	15 (75%)	
High	14 (18.9%)	5 (25%)	
Total protein, n (%)			0.016
Low	52 (71.2%)	9 (42.9%)	
Normal	21 (28.8%)	12 (57.1%)	
Albumin, n (%)			0.123
Low	27 (37%)	4 (19%)	
Normal	46 (63%)	17 (81%)	
Direct bilirubin, n (%)			0.148
Normal	50 (69.4%)	11 (52.4%)	
High	22 (30.6%)	10 (47.6%)	
Creatinine, n (%)			0.453
Normal	62 (86.1%)	16 (76.2%)	
High	10 (13.9%)	5 (23.8%)	
Uric acid, n (%)			0.465
Normal	54 (74%)	17 (85%)	
High	19 (26%)	3 (15%)	
K, n (%)			0.133
Low	16 (22.2%)	1 (4.8%)	
Normal	56 (77.8%)	20 (95.2%)	
ACTH, n (%)			0.853
Normal	50 (82%)	14 (82.4%)	
Low	9 (14.8%)	2 (11.8%)	
High	2 (3.3%)	1 (5.9%)	
Renin (standing), n (%)			0.872
High	14 (20.6%)	4 (21.1%)	
Normal	43 (63.2%)	11 (57.9%)	
Low	11 (16.2%)	4 (21.1%)	
Renin (decubitus), n (%)			0.189
Normal	50 (72.5%)	10 (52.6%)	
High	4 (5.8%)	3 (15.8%)	
Low	15 (21.7%)	6 (31.6%)	
Aldosterone (standing), n (%)			0.367
Normal	62 (89.9%)	17 (81%)	
High	6 (8.7%)	4 (19%)	
Low	1 (1.4%)	0 (0%)	
Aldosterone (decubitus), n (%)			0.906
High	11 (15.7%)	2 (11.1%)	
Normal	59 (84.3%)	16 (88.9%)	
Tumor Size, median (IQR)	20 (13, 30)	32.5 (20, 50)	0.009

### 3.3 Univariate analysis and multivariate analysis

Univariate analysis identified low TP (OR=0.303, 95% CI=0.111-0.825, P=0.020) and tumor size (OR=1.045, 95% CI=1.011-1.080, P=0.009) as potential risk factors for malignant AI in multivariate analysis (**Table 3**). Multivariate analysis further confirmed that low TP and larger tumor size were significant independent risk factors for malignant AI (**Table 3**).

**Table 3:** Univariate analysis and multivariate analysis.

	Univariate logistic analysis			Multivariate logistic analysis		
	OR	95%CI	p value	OR	95%CI	p value
low Total Protein	0.303	0.111-0.825	0.0195	0.225	0.068-0.748	0.0195
Tumor Size	1.045	1.011-1.080	0.0088	1.045	1.010-1.081	0.0115

#### 3.3.1 Development of an individualized prediction nomogram

**Figure 2:**
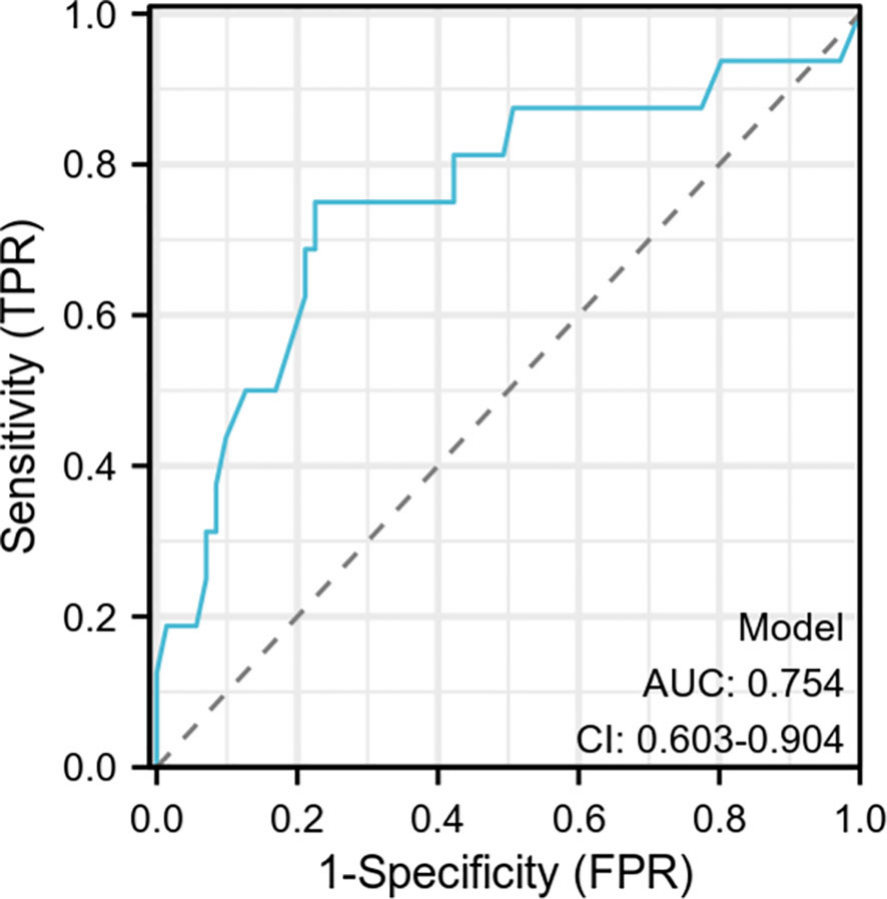
Specificity and sensitivity to distinguish benign and malignant AI model.

A predictive model incorporating clinical risk factors, specifically tumor size and low TP, was established. The model demonstrated an Area Under the Curve (AUC) of 0.754 (95% CI, 0.603-0.904) with a sensitivity of 0.75 and specificity of 0.775. The calibration curve, showing a bias-corrected AUC of 0.77, is presented in **Figure 2**, along with the ROC curve.

The model demonstrated an AUC of 0.754 (95% CI, 0.603-0.904) with a sensitivity of 0.75 and a specificity of 0.775. The calibration curve showed a bias-corrected AUC of 0.77.

## 4 Discussion

We are committed to discovering the impact of the COVID-19 infection on AIs. Through our statistical analysis, we found that there are certain differences in obesity and direct bilirubin in AI after explosive infection with the COVID-19 compared with before. However, after univariate analysis and multivariate analysis, we found no significant risk factors.

There are currently many studies on the relationship among obesity, COVID-19 and AI. British researchers believe obesity is closely related to the stay-at-home lockdown during the COVID-19 pandemic (23). But some researchers have found that obesity contributes to increased complications/severity of COVID-19 and changes in mortality rates (24). In addition, researchers have found that among obese patients who died from COVID-19, the virus easily spreads to endocrine organs (25). Regarding the relationship between obesity and AI, interestingly, recent studies have shown that patients with AIs are more likely to be obese and insulin resistant (26). Additionally, AI patients may show elevated levels of adipokines (apparently not related to diabetes, hypertension, or obesity), which may be influenced by the presence of adrenal adenomas (27). Obesity is related to COVID-19 and AIs, but current research does not provide a cause-and-effect relationship. In our study, we found that patients with AIs suffering from COVID-19 had a higher proportion of obesity, which is also consistent with the conclusions of the current study. But further experiments may be needed to verify the relationship.

There are many studies on the relationship between bilirubin levels and the severity and prognosis of COVID-19. Most of them believe that high levels of direct bilirubin will lead to worse prognosis and higher fatality rate of COVID-19 (28–31). Some researchers have also found that direct bilirubin levels affect the severity and prognosis of COVID-19 (29, 31). And direct bilirubin levels are more likely to be abnormal after COVID-19 infection (32). We found no studies related to bilirubin and AI, so we believe that COVID-19 infection is the main cause of direct bilirubin elevation.

Literatures shows that the incidence of AI after the epidemic is more than 10 times that before the epidemic. Most researchers believe that it is related to the routine chest X-ray examination during the epidemic, because chest X-rays can detect part of the adrenal glands. After our data analysis, there was no statistically significant decrease in the diagnosis rate/incidence rate of AI compared with before the COVID-19 outbreak (4.14% vs 4.73% P=0.453). There was no statistically significant increase in the incidence of adrenal nonfunctioning adenoma (42.1% vs 33.3% P=0.322). In the process of decreasing the incidence of AI after COVID-19 infection, we found that the incidence of NFAI increased after COVID-19 infection. Although there is no significant difference in the results, we still believe that the COVID-19 causes hyperplasia of the adrenal cortex and ultimately leads to adrenal incidentalomas. Our negative results may be caused by the following reasons: First, this study was conducted within a short period of time after COVID-19 infection and failed to show its specificity. Second, when faced with small-volume non-functioning adrenal incidentalomas, clinicians generally adopt conservative treatment. This treatment option will cause us to miss data on some patients. Third, the data from a single center with a small sample has certain result bias. We believe that it may take a longer time for a transient increase in Ang II to manifest its significance.

In our investigation into primary malignancies among adrenal incidentalomas (AIs), we identified significant associations with total protein levels and tumor size. Notably, lower total protein levels and smaller tumor size in AIs were indicative of lower malignant potential. This finding aligns with existing research suggesting that serum total protein levels hold diagnostic significance, facilitating discrimination between cancer patients and healthy individuals. Specifically, some scholars propose the utility of total protein as an adjunct diagnostic marker for malignancy detection (33).

Moreover, the relevance of total protein extends beyond diagnosis, as evidenced by studies linking total protein values to higher in-hospital mortality rates among general inpatients. This association persists across patients both with and without malignancy (34). Additionally, the level of total protein emerges as a critical factor influencing the prognosis of various cancers, including lung cancer, stomach cancer, and liver cancer. Furthermore, total protein levels are implicated in chemotherapy outcomes, underscoring their multifaceted role in cancer-related prognostication and treatment response (35–38). These findings collectively highlight the potential of total protein as a versatile biomarker with implications for both diagnosis and prognostication in the context of adrenal incidentalomas and malignancies.

Some researchers believe that for AI, there is no clear line between NFAI and biochemically functional AI. In other words, NFAI can progress over time and transform into secretory AI. It is generally believed that 3-4cm is an obvious dividing line, that is, if the diameter of AI is greater than 3 or 4cm, then the possibility of them having secretory function and tending to be malignant is greatly increased. However, there is currently no clear conclusion on whether 3cm or 4cm is the limit (39). Therefore, we can believe that as the tumor size increases, the malignancy of AIs will also increase, which is also consistent with the results of the current study.

In addition, some researchers found that the functionality of AI is related to the neutrophil/lymphocyte ratio, but in our study, no correlation was found (40).

Therefore, we have reasons to believe that the malignant potential of AI with larger tumor diameters and concurrent lower than normal total protein values is more obvious.

## 5 Conclusion

We were unable to find differences in the clinical manifestations of adrenal incidentalomas caused by COVID-19 infection. And we found that AI with larger tumor diameters and lower than normal levels of total protein had a more significant malignant potential.

## Data Availability

The raw data supporting the conclusions of this article will be made available by the authors, without undue reservation.
